# Novel Antibacterial, Cytotoxic and Catalytic Activities of Silver Nanoparticles Synthesized from Acidophilic Actinobacterial SL19 with Evidence for Protein as Coating Biomolecule

**DOI:** 10.4014/jmb.2205.05006

**Published:** 2022-08-22

**Authors:** Magdalena Wypij, Maciej Ostrowski, Kamil Piska, Katarzyna Wójcik-Pszczoła, Elżbieta Pękala, Mahendra Rai, Patrycja Golińska

**Affiliations:** 1 Department of Microbiology, Nicolaus Copernicus University, Torun 87-100, Poland; 2 Department of Biochemistry, Nicolaus Copernicus University, Torun 87-100, Poland; 3 Department of Pharmaceutical Biochemistry, Faculty of Pharmacy, Jagiellonian University Medical College, Medyczna 9, 30-688, Kraków, Poland; 4 Nanobiotechnology Laboratory, Department of Biotechnology, SGB Amravati University, Amravati 444602, India

**Keywords:** Acidophilic actinomycetes, antibacterial and anticancer activity, capping agent, catalytic activity, green synthesis, nanosilver

## Abstract

Silver nanoparticles (AgNPs) have potential applications in medicine, photocatalysis, agriculture, and cosmetic fields due to their unique physicochemical properties and strong antimicrobial activity. Here, AgNPs were synthesized using actinobacterial SL19 strain, isolated from acidic forest soil in Poland, and confirmed by UV-vis and FTIR spectroscopy, TEM, and zeta potential analysis. The AgNPs were polydispersed, stable, spherical, and small, with an average size of 23 nm. The FTIR study revealed the presence of bonds characteristic of proteins that cover nanoparticles. These proteins were then studied by using liquid chromatography with tandem mass spectrometry (LC-MS/MS) and identified with the highest similarity to hypothetical protein and porin with molecular masses equal to 41 and 38 kDa, respectively. Our AgNPs exhibited remarkable antibacterial activity against *Escherichia coli* and *Pseudomonas aeruginosa*. The combined, synergistic action of these synthesized AgNPs with commercial antibiotics (ampicillin, kanamycin, streptomycin, and tetracycline) enabled dose reductions in both components and increased their antimicrobial efficacy, especially in the case of streptomycin and tetracycline. Furthermore, the in vitro activity of the AgNPs on human cancer cell lines (MCF-7, A375, A549, and HepG2) showed cancer-specific sensitivity, while the genotoxic activity was evaluated by Ames assay, which revealed a lack of mutagenicity on the part of nanoparticles in *Salmonella Typhimurium* TA98 strain. We also studied the impact of the AgNPs on the catalytic and photocatalytic degradation of methyl orange (MO). The decomposition of MO was observed by a decrease in intensity of absorbance within time. The results of our study proved the easy, fast, and efficient synthesis of AgNPs using acidophilic actinomycete SL19 strain and demonstrated the remarkable potential of these AgNPs as anticancer and antibacterial agents. However, the properties and activity of such particles can vary by biosynthesized batch.

## Introduction

The biosynthesis of silver nanoparticles (AgNPs) using microorganisms such as bacteria, fungi, cyanobacteria, actinomycetes, and algae offers low production costs and high efficiency [1]. Biomolecules derived from microorganisms have been successfully utilized for the reduction of metal ions to nanoparticles (NPs), as in the case of Ag^+^ [2, 3]. In the green chemistry of AgNPs, biomolecules are also involved in the coating of NPs [3], thereby preventing their agglomeration which significantly decreases the bioactivity and cell association that is crucial in medical applications [4, 5]. It is believed that biogenic AgNPs are biocompatible due to natural capping, which is an advantage compared to physically and chemically produced AgNPs [1]. However, the coating of biomolecules such as proteins may influence the antimicrobial and cellular cytotoxicity exerted by AgNPs [6, 7]. Such proteins can induce various biological reactions which in turn can influence physicochemical and cellular mechanisms in the host cells. This is an important issue regarding the safe use of nanoparticles as antimicrobial agents [6]. Unfortunately, so far, the coating of bio-nanoparticles has not been comprehensively studied.

Nowadays, the growing resistance of pathogenic microorganisms to antibiotics is a serious threat to human health and other living beings [8]. Therefore, AgNPs are considered promising agents in the fight against pathogens, including multidrug-resistant (MDR) bacteria, as they rarely develop resistance to AgNPs that have several targets in microbial cells [8]. As reported previously, AgNPs can efficiently eliminate a variety of bacterial and fungal pathogens, such as *Escherichia coli*, *Klebsiella pneumoniae*, *Staphylococcus aureus*, *C. albicans*, and *A. niger*, as well as viruses (HBV and HIV) [9], even at very low concentrations [9]. In addition, their antibiofilm properties and synergistic effect with antimicrobials against pathogenic bacteria and fungi have also been reported [10, 11].

Currently, scientists around the world are looking for new and effective therapies against cancer, which is a leading cause of death worldwide [12]. Many in vitro and in vivo studies indicated that AgNPs might be used as a promising anti-cancer agent against cervical, breast, lung, hepatitic, nasopharyngeal, glial, colorectal, and prostate cancer cells [13-16].

The present study is a continuation of our investigations on biogenic AgNPs synthesized from actinomycetes in general and acidophilic strain in particular [17, 18] and was designed to establish the potential use of such AgNPs in biomedical applications as antibacterial, antibiofilm, and anticancer agents. For this purpose, we determined the standard minimum inhibitory and biocidal concentrations of AgNPs, the growth kinetics of bacteria in the presence of AgNPs, the fractional inhibitory index of AgNPs with commercial antibiotics and finally the antibiofilm properties. Moreover, the catalytic activity and physicochemical properties of biosynthesized AgNPs were characterized. The protein capping was studied to understand its correlation with bioactivity and biocompatibility. Cytotoxic activity was tested on four cancer cell lines.

It should be emphasized that in this study AgNPs were synthesized from an acidophilic actinomycete strain belonging to the rare genus *Pilimelia*, as identified previously [17].

Although the biosynthesis of silver nanoparticles from microorganisms, algae, and plants is a well-developed procedure, the use of acidophilic actinobacteria for this process is still in its infancy [19, 20]. In general, acidophilic/acidotolerant actinomycetes are less studied when compared to their neutrophilic counterparts, but they possess large genomes encoding for the synthesis of bioactive compounds and enzymes [21] that can be involved in an electron shuttle in metal ion reduction and/or formation of capping agents for NPs [22, 23]. Therefore, in our study on the biosynthesis of AgNPs we paid particular attention to such a group of microorganisms. In addition, biogenic synthesis by actinomycetes is an eco-friendly, easy, and cost-effective approach [22, 24].

## Materials and Methods

### Synthesis of Silver Nanoparticles by Acidophilic Actinomycete Strain SL19

The biomass of acidophilic actinomycete strain SL19 used for the production of AgNPs was obtained by its growth in International *Streptomyces* Project 2 (ISP2) broth [25] at pH 5.5. The culture was grown under controlled conditions in an orbital shaker (120 rpm) for one week at 27°C, and then centrifuged at 6,000 ×*g* for 10 min. The biomass was washed with sterile distilled water, resuspended in sterile water, and incubated for 3 days at 27°C for autolysis. Finally, the autolysate was centrifuged (6,000 ×*g* for 10 min) to eliminate cell debris while the supernatant was combined with silver nitrate (final concentration 1 mM) and incubated for 3 days at 27°C in the dark. The green synthesis of AgNPs was recorded by visual observation of the color change of the reaction mixture from yellow to brown.

### Characterization of AgNPs

**Spectroscopic Analyses-UV-Visible Spectroscopy**

The presence of biogenic nanosilver was confirmed by analysis of the surface plasmon resonance band using UV-Vis spectroscopy (NanoDrop ND2000, Thermo Scientific, USA). The absorption spectrum of the sample was recorded in the range of 200-800 nm, at a resolution of 1 nm.

### Fourier-Transform Infrared (FTIR) Spectroscopy

Spectra were recorded using an FTIR spectroscope (Spectrum 2000, Perkin-Elmer, USA) with a range between 400–4,000 cm^-1^ and a diffuse reflectance accessory. The AgNPs (1%, w/w) were mixed with potassium bromide (KBr) prior to measurements.

### Transmission Electron Microscopy (TEM)

TEM (FEI Tecnai F20 X-Twintool, USA) at a voltage of 100 kV was used for analyses of the size and shape of nanoparticles. The colloidal suspension of AgNPs in deionized H_2_O was drop deposited on carbon-coated copper (400 mesh size) grids.

### Zeta Potential

The zeta potential of AgNPs was measured by using a Zetasizer (ZS 90, UK). The nanoparticles were sonicated for 15 min at 20 Hz to avoid aggregation and diluted to a final concentration of 100 μg/ml prior to measurement.

### X-Ray Diffraction (XRD) Spectroscopy Analysis

The structure of the AgNPs was examined by X-ray diffraction analysis. The XRD patterns were recorded over a 2 h range of 5°–120° by an X-ray diffractometer (X’Pert Pro, Philips Analytical, The Netherlands) equipped with Ni filter and CuKa (k = 1.54056 Ao) radiation source.

### Analyses of Proteins Coated on AgNPs

**Preparation of Samples, SDS-PAGE, and Identification of Proteins**

For extraction of proteins the biomass of strain SL19 was washed 5 times with sterile distilled water, suspended in 50 ml of a Tris-HCl buffer (50 mM, pH 8.0) with NaCl (300 mM), glycerol (10%, v/v), and EDTA (1 mM), sonicated for 20 s at 20 kHz using an ultrasonic homogenizer (Omni Ruptor, Omni Int., USA) and centrifuged at 10,000 ×*g* for 15 min. The supernatant was combined with silver ions and the concentration of proteins in the mixture was evaluated using the Bradford method [26]. The control sample was the supernatant without silver ions.

The SDS-PAGE was performed in polyacrylamide gel (10 and 4 % (w/v) running and stacking gels, respectively) at a voltage of 150 V for 2 h [27]. Wells of the gel were loaded with samples containing 20 μg of proteins. The polyacrylamide gel was stained with Coomassie Brilliant Blue dye [28]. The separated protein bands were cut off from gel and proteins were digested with trypsin prior to analysis by LC-MS/MS (nanoAcquity UPLC Waters, Etten-Leur, The Netherlands; Orbitrap Velos mass spectrometer, Thermo Scientific) at the Institute of Biochemistry and Biophysics, Polish Academy of Sciences in Warsaw, Poland. The obtained data were analyzed by the MASCOT program.

### Assessment of AgNP Antimicrobial Activity

**Minimal Inhibitory Concentration (MIC) Determination**

MICs of AgNPs and antibiotics (ampicillin, streptomycin, kanamycin, and tetracycline) were determined using the microdilution method according to Clinical and Laboratory Standards Institute (CLSI) guidelines [29] against *Staphylococcus aureus* ATCC 6538, *E. coli* ATCC 8739, *K. pneumoniae* ATCC 700603, and *Pseudomonas aeruginosa* ATCC 10145. The analysis was performed in 96-well plates using serial two-fold dilutions of AgNPs or antibiotics in Tryptic Soy Broth (TSB; Becton Dickinson, USA) at concentrations ranging from 0.125 to 2,048 μg/ml. The final bacterial concentration in each well was 1.5 × 10^6^ CFU/ml. Inoculated plates were incubated for 24h at 37°C. The TSB medium without tested bacteria was a negative control while the inoculated broth was a positive control. The MIC endpoint was determined as the lowest concentration of AgNPs or antibiotics at which the visible growth of the bacterium was inhibited after incubation time.

### Minimal Biocidal Concentration (MBC) Determination

The MBC of AgNPs and antibiotics was determined after spreading 0.1 ml of tested samples from the above experiment on Tryptic Soy Agar (TSA; Becton Dickinson) plates and incubating them for 24 h at 37°C. The MBC endpoint was determined as the concentration of antimicrobial agent that inhibited ≥ 99.9% of the bacterial population.

### Tolerance Level of Bacterial Pathogens to AgNPs

The tolerance level of bacteria to AgNPs was determined according to the following formula: T = MBC/MIC, where T is tolerance [30].

The ratio of MBC/MIC ≥16 indicates the bacteriostatic action of AgNPs, while ≤ 4 indicates the bactericidal action of AgNPs [31].

### Determination of Fractional Inhibitory Concentration Index (FICI)

The combined effect of biogenic AgNPs and antibiotics like streptomycin, kanamycin, ampicillin, and tetracycline was determined using the fractional inhibitory concentration index [32]. The FICI was calculated according to the following formula: FICI = (MIC of AgNPs in combination with antibiotic)/(MIC of AgNPs) + (MIC antibiotic in combination with AgNPs)/MIC of antibiotic).

### Determination of Growth Curve

The effect of biogenic AgNPs on the growth kinetics of bacteria was monitored by optical density measurements. The growth curves of each bacterial culture at ½, 1 ×, and 2 × MICs of nanoparticles were generated based on the mean value from three replicates. Analyses were performed in sterile 96-well plates. The final concentration of bacterial cells used in wells was 1.5 × 10^6^ CFU/ml. Wells containing non-inoculated media were taken as negative control samples while the inoculated ones were used as positive control samples. The inoculated plates were incubated for 24 h at 37°C using a plate reader (Multidetection Spectramax iD3, Molecular Devices, USA) and read for OD value at a wavelength of 580 nm at 2 h intervals.

### Biofilm Formation Assay

The influence of biogenic AgNPs on biofilm formation by test bacteria was investigated using 96-well, flat-bottom plates. The AgNP concentrations used in this experiment were from 1/512 to 2 × MIC. The multi-well plates were inoculated with bacteria (final inoculum concentration was 1.5 × 10^6^ CFU/ml) and kept for 24 h at 37°C for biofilm development. The medium above the biofilm layer was then removed gently and wells were washed three times with sterile distilled water to eliminate free bacterial cells. The plates were dried for 60 min at room temperature; the biofilm was stained with 1% (w/v) crystal violet for 15 min and then washed three times with sterile distilled water to remove the non-absorbed dye. Subsequently, 200 μl of 96% (v/v) ethanol was added to each well to elute absorbed dye from the biofilm. The absorbance value of the released dye was measured at 595 nm using a plate reader (Multidetection Spectramax iD3, Molecular Devices). Negative (non-inoculated broth) and positive (inoculated broth) controls were also maintained. The % of biofilm inhibition was calculated according to the following formula:

[1 – OD_595_ of treated cells /OD_595_ of non-treated cells (control)] × 100.

### Cytotoxicity Test of AgNPs

**Cell Cultures**

Breast cancer (MCF-7), human melanoma (A375), adenocarcinomic human alveolar basal epithelial (A549), and liver hepatocellular carcinoma (HepG2) cell lines were acquired from American Type Culture Collection (ATCC). The MCF-7, A375, and A549 cell lines were cultured in Dulbecco’s modified Eagle medium (DMEM, Sigma-Aldrich, Germany) while HepG2 was cultured in Eaglés minimum essential medium (EMEM, Sigma-Aldrich,) and both were supplemented with a 10% FSB (fetal bovine serum and penicillin/streptomycin antibiotic mixture (1%, w/v) purchased from Gibco (USA). Cultures were grown in standard conditions of temperature (37°C) and CO_2_ concentration (5%).

### Viability Assays

Cells (5-10 × 10^3^ per well) were seeded into the proper medium in 96-well plates for 24 h to form a monolayer. Then, cells were treated with AgNPs at concentrations ranging from 0.1 to 100 μg/ml for 48 h, and viability assays (MTT and NRU) were subsequently performed.

### MTT Test

The cell lines after incubation with AgNPs were washed with 200 μl of phosphate-buffered saline (PBS) before being treated with 3-(4,5-dimethylthiazol-2-yl)-2,5-diphenyltetrazolium bromide (Sigma-Aldrich) (5 mg/ml) for 3 h. The formed formazan was dissolved in 100 μl of dimethyl sulfoxide (DMSO; Sigma-Aldrich) that was added to plate wells. The absorbance of the sample at 570 nm was then measured using a microplate reader (Spectra Max iD3, Molecular Devices). The cell viability (V) was calculated according to the following formula: V= absorbance of experimental well/absorbance of control well (non-treated) × 100%. Three separate experiments were performed, each in triplicate.

### Neutral Red Uptake (NRU) Assay

Neutral red dye was applied to evaluate cell lysosome functionality. The culture medium was exchanged for medium with the addition of neutral red (40 μg/ml) after incubation in the presence of AgNPs. The plates were incubated for 2 h and then cells were washed two times with PBS (pH 7.4, 6.7 mM PO_4_) and destained with 100 μl of a solution containing 1% glacial acetic acid, 50% EtOH, and 49% H_2_O. Once intracellular NR dye was dissolved in the destaining solution the absorbance was measured at 540 nm using a plate reader (Spectra Max iD3, Molecular Devices, USA). The cell viability was determined according to the formula given in the MTT assay.

### ROS Generation

The generation of ROS was analyzed by using CM-H2DCFDA (non-fluorescent dye) which is intracellularly activated and under oxidation conditions chemically converted to a highly fluorescent agent. Therefore, the intensity of fluorescent light from cells is related to redox imbalance in cells [33]. This assay was performed on A549, MCF-7, and HepG2 cell lines. The A375 cell line was excluded from the analysis due to autofluorescence properties. Cell lines were seeded into transparent, black, 24-well plates (Germany) at a density of 25 × 10^3^ per well for 24 h. Cells were then loaded with CM-H2DCFDA (5 μM; Sigma-Aldrich, Germany) for 60 min, rinsed two times with 0.5 ml PBS (pH 7.4, 6.7 mM PO_4_) and treated with FluoroBrite DMEM (Sigma-Aldrich) containing AgNPs (0.1, 1, 10, 25, 50, 75, and 100 μg/ml), H_2_O_2_ (0.03 % v/v) or control (water). Then, the cells were observed under a fluorescence microscope (Leica DMIL LED Fluo, Leica Microsystems GmbH, Germany) for 180 min in five randomly selected fields. Microphotographs were taken using LAS-X Software (Leica Microsystems GmbH). All analyses were performed in triplicate.

### Ames Test

*Salmonella Typhimurium* strain TA98 (Trinova Biochem GmbH, Germany) was used for the genotoxicity assay. The tested strain was grown in nutrient broth No. 2 (Oxoid) for 24 h at 37°C. Initially, the aliquots (100 μl) of overnight bacterial culture (1.5 × 10^8^ CFU/ml) were mixed 1:1 (v/v) with AgNPs at various concentrations (0.15, 0.25, 0.5, 1.0, 1.5, 3.0, 6.0, 12.5, 25.0, 37.5, 50.0, and 100.0 μg/plate) under shaking conditions (80 rpm) for 2 h at 37°C [34]. The samples were then mixed with 2 ml of molten top agar (Trinova Biochem GmbH), poured as a second layer on Petri plates with minimum salt agar (Trinova Biochem GmbH), and incubated for 48 h at 37°C. The positive control contained bacteria treated with 2-nitrofluorene (Sigma-Aldrich) at 3 μg/plate. Non-treated bacteria were used as a negative control. All analyses were performed in triplicate.

### Catalytic Degradation of Organic Dye

The catalytic reduction of methyl orange (MO, 0.1 mg/ml) with 0.1 M sodium borohydride (NaBH_4_) as a reducing agent was studied at different time points using UV–Vis spectroscopy. Two sets of reactions were performed:

1) the dye solution was treated with sodium borohydride (blank experiment);

2) the dye solution was treated with sodium borohydride and different concentrations of AgNPs (8, 16, 32, 64, 128, 256, and 512 μg/ml). The absorbance was analyzed at a wavelength of 460 nm for 10 min at an interval of 1 min at room temperature.

### Catalytic Activity of AgNPs

The catalytic activity (in the presence of sunlight) of AgNPs at different concentrations, namely 32, 64, and 256 μg/ml, was evaluated for degradation of methyl orange (MO) aqueous solutions at 0.1 mg/ml after 1, 10, and 60 min and 3.5 h by using UV-Vis spectroscopy (NanoDrop ND2000, Thermo Scientific) in the range of 200-800 nm. The silver nanoparticles in specific concentrations were added to the MO solution. The dye solution without silver nanoparticles added was maintained as a control.

### Statistical Analysis

The TEM data were analyzed using Statistica software (StatSoft Inc., USA). Statistical analysis for cytotoxicity data was performed in Origin 2021. One-way ANOVA followed by post hoc analysis (Tukey’s test) was used to determine statistical significance at *p* < 0.05 for cytotoxicity analyses.

## Results and Discussion

The resistance of pathogenic bacteria to antimicrobial agents has been growing significantly in recent years [35]. Similarly, the number of cancer cases, especially breast cancer, followed by colorectal cancer, and related mortality have also been increasing worldwide [36-38]. Thus, nanoparticles may provide an alternative to challenge antibiotic resistance in microorganisms [39] and are increasingly used for the diagnosis and therapeutics of cancer-related diseases [40].

The traditional chemical and physical methods that are most often used for AgNPs synthesis include the use of harsh, environmentally degrading chemical reagents and procedures that are too expensive to be feasible on an industrial scale [41]. For these reasons, the interest in green chemistry to produce nanoparticles with desired properties has been increasing in recent years [42].

### Synthesis and Physicochemical Characterization of AgNPs

In the present study, the biogenic synthesis of AgNPs using an actinomycete strain was found to be fast, easy, inexpensive, and efficient. The process was monitored through the change in the color of the reaction mixture from colorless to brown. The UV-Vis spectroscopy of the sample, which revealed a peak at 421 nm and is characteristic of AgNPs [7], confirmed their presence in the reaction solution (Fig. S1). The complex characterization of the physicochemical properties of AgNPs is important for their effective and safe application in the future [43].

The AgNPs from strain SL19 examined by TEM were polydispersed, small in size (mean of 23 nm) well dispersed, and spherical (Fig. S2). Most of the previously published reports on the synthesis of AgNPs using actinomycetes showed the fabrication of small (4-85 nm), spherical, and polydispersed nanoparticles [17, 34, 44].

X-ray diffraction analysis showed diffraction peaks at = 32.5°, 48.4°, 68.2°, and 78.6° and confirmed the crystalline nature of synthesized nanoparticles (Fig. S3). These peaks might be assigned to the planes (111), (200),(220), and (311) facets of a silver crystal, respectively. An unassigned peak present in the sample could be due to the presence of bio-organic molecules on the surface of the AgNPs, as suggested by Ahmad and Sharma [44]. A similar peak pattern has been found in AgNPs from the acidophilic actinomycete strain reported previously by Wypij [7].

The zeta potential of AgNPs synthesized by actinomycete SL19 strain was equal to -21.4 mV, as shown in Fig. S4. This charge value results from the occurrence of numerous negatively charged functional groups in the nanoparticle coating [45] and indicates the stability of the obtained AgNPs. The closer the nanoparticle surface charge is to -30 mV, the more stable the nanoparticles are in the solution [45,46]. Similar zeta potential values for AgNPs synthesized by actinomycetes were also reported previously [7, 10, 17, 47].

**Fig. 1:**
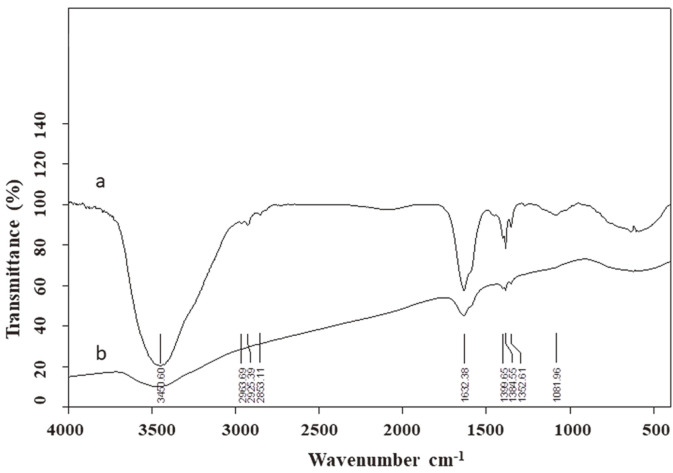
FTIR spectrum of biosynthesized AgNPs. AgNPs (a) and control (b).

The FTIR analysis (Fig. 1) showed that the examined AgNPs exhibit an absorbance band at the length of 3450.6 cm-1, which is characteristic of O-H hydrogen bonds or N-H stretching bonds of primary amines [48]. The bands at 2963.69, 2925.39, and 2853.11 cm^−1^ are attributed to C–H (alkane) stretching bonds [49]. The band at 1638.38 cm^−1^ corresponds to the amide groups of proteins which could be released by aromatic carbon compounds and carbon double bonds as well as alkene groups (-C–H stretch) [50], while these at 1399.65, 1384.55 and 1352.61 cm^−1^ are amide peaks [51]. Moreover, the peak found at 1081.96cm^−1^ can be assigned to the C–N bonds of the amine [52]. The functional groups like the hydroxyl and carbonyl ones on the surface of the bionanoparticles indicate molecules such as amino acids and/or proteins that can be involved in a reduction of Ag^+^ to Ag^0^ [16] and the formation of AgNPs [53]. It is interesting that the size, zeta potential, and FTIR pattern for AgNPs from SL19 are similar to those previously found [17], but are not identical. It is clear that the biogenic synthesis process of AgNPs needs further optimization.

### Analysis of Protein Coating on AgNPs

**Fig. 2:**
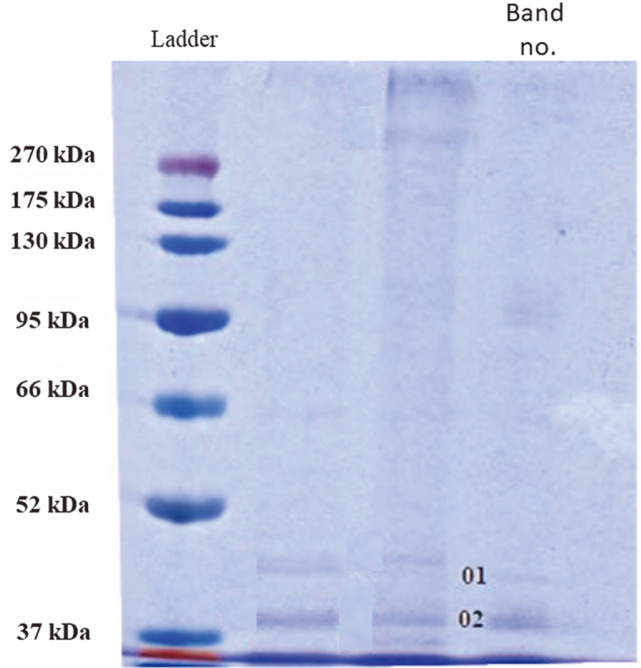
SDS-PAGE of proteins associated with biosynthesized AgNPs.

Protein capping of AgNPs strongly binds silver ions through the free amino acids, but mainly by cysteine residues, and are responsible for nanoparticle stabilization in the solution preventing the formation of aggregates [53, 54]. The analysis of the protein coating of AgNPs from strain SL19 showed the presence of hypothetical protein (01) and porin (02) (59% cover) with molecular masses of 41 and 38 kDa (Fig. 2) and an isoelectric point (pI) of 8.47 and 8.81, respectively (Table S1), that were absent in the control filtrate. It should be emphasized that, besides our group, the previous reports have mainly focused on the analysis of proteins engaged in the synthesis and/or capping of AgNPs derived from fungi. However, not all of these reports have provided protein identification, as discussed below. Rodrigues and coauthors [55] reported that the protein bands (75, 122, 191, and 328 kDa) occurring in the fungal filtrate were found in the coating of AgNPs produced by *Aspergillus tubingensis*. Similarly, Jain *et al*. [56] noticed that the filtrate of *Aspergillus flavus* used for biosynthesis contained two protein bands of 32 and 35 kDa that were later found in the AgNPs’ coating. In other studies, Guilger-Casagrande *et al*.[57], obtained bands of 36 and 40 kDa, both found in the filtrate and capping samples. The molecular weights of these proteins corresponded to β-1,3-glucanase and chitinase from *Trichoderma harzianum*. It is claimed that the coating composition of nanoparticles may determine their biocompatibility and provide an active surface for interaction and/or conjugation with variable bioactive substances including anticancer and antimicrobial drugs [57, 58].

### Assessment of AgNP Antimicrobial Activity

**Determination of Minimal Inhibitory and Minimal Biocidal Concentrations**

In this study, biosynthesized AgNPs displayed antimicrobial properties against all tested strains. However, gram-negative bacteria were more sensitive than those that are gram-positive. This tendency was not found in the previously published studies on AgNPs from SL19 strain [17] where MIC and MBC values against these bacterial strains were determined in the range of 70-90 and 100 to >200 μg/ml, respectively [17]. In the present study, the MIC values of Ag nanoparticles for *K. pneumoniae* and *S. aureus* were 256 μg/ml, while for *P. aeruginosa* and *E. coli*, they were 32 and 64 μg/ml, respectively. The MBC values of AgNPs for *E. coli*, *K. pneumoniae*, and *P. aeruginosa* were equal to the corresponding MIC values while for *S. aureus* they were found to be 512 μg/ml (Table 1). Divergency in the activities of AgNPs from SL19 strain against bacteria may result from differences in their properties found in each synthesized sample, as mentioned above. Moreover, different assays were used to determine these activities. Overall, *S. aureus* was most sensitive to all tested antibiotics (MICs ranged from 0.016 to 4 μg/ml) in contrast to *K. pneumoniae* (MICs ranged from 4 to 2,048 μg/ ml). Similar correlations have been found in the case of MBCs of antibiotics (Table 1). Al-Dhabi *et al*. [59] studied AgNPs from marine-derived actinomycetes that exhibited significantly higher antibacterial activity against gram-negative bacteria, namely *E. coli* (31.25 μg/ml), *K. pneumoniae* (62.5 μg/ml) and *P. aeruginosa* (15.6 μg/ml) than gram-positive bacteria like *Enterococcus faecalis* (125 μg/ml), *Bacillus subtilis* (250 μg/ml) and *Staphylococcus epidermidis* (250 μg/ml), which supports the tendency found in our studies. In contrast, Mondéjar-López *et al*. [60] reported that the MICs of biogenic AgNPs from *Iris tuberosa* against *P. aeruginosa* and *S. aureus* (14.1 μg/ml) were much lower than those against *E. coli* (44.4 μg ml^-1^). Meanwhile, Rautela *et al*. [61] reported that the MICs of bio-AgNPs from Tectona grandis seed extract against Bacillus cereus, *S. aureus*, and *E. coli* were found to be 5.2, 2.6, and 2.0μg/ml, respectively.

### Tolerance Level and Growth Curve of Bacterial Pathogens in the Presence of AgNPs

The tolerance level of *S. aureus*, *K. pneumoniae*, *E. coli*, and *P. aeruginosa* to bionanoparticles was estimated as 2, 1, 1, and 1, respectively. Tested antibiotics revealed the lowest tolerance of bacteria to tetracycline and the highest to ampicillin and kanamycin (Table S2).

Analyses of growth curves of bacteria under AgNP treatment at corresponding MICs showed that gram-negative and gram-positive bacteria were strongly inhibited throughout the experiment (Fig. S5). Even a concentration of 1/2 MIC of AgNPs essentially inhibited the growth of gram-negative bacteria in the analyzed duration time. Although the antibacterial activity of AgNPs is well reported, the specific mechanism of their action has not been completely explained. It is claimed that the biocidal properties of AgNPs are mostly related to direct action on the cell envelopes (cell wall and membrane), followed by penetration of the cell membrane into cytosol and disintegration of the membrane structure. This leads to the loss of cell components and cell death [62]. However, the release of silver ions from nanosilver is claimed to be a key mechanism responsible for its biocidal action [63]. According to Khorrami *et al*. [64], the Ag^+^ ions, like AgNPs, adhere to the cell wall and membrane and significantly enhance the permeability of the latter which results from electrostatic attraction and the strong affinity of ions for sulfur proteins.

### Determination of Fractional Inhibitory Concentration Index (FICI)

This study showed a significant synergistic effect resulting from the combination of nanoparticles with selected antibiotics (Table 2). This action allowed for a significant reduction in the concentration of antibiotics and nanoparticles. For example, combined use of streptomycin with AgNPs was reduced by 4, 8, and 32 times the dose of both antimicrobials against *E. coli*, *P. aeruginosa*, and *K. pneumoniae*, respectively, when compared to compounds used separately. Similarly, the use of tetracycline and AgNPs reduced by eight times the doses of both compounds when evaluated against *E. coli* and *P. aeruginosa*, and 16 times against *K. pneumoniae* (Table 2). Although the molecular mechanism of synergism is not fully understood [65], undoubtedly, such combined therapy is beneficial as it increases the effectiveness of antibiotics and minimizes the duration of treatment and the dose of antibiotics [66]. This could also greatly contribute to solving the problem of antibiotic resistance and decreasing the toxicity of nanosilver towards mammalian cells [67, 68]. The phenomenon of synergism is probably related to the formation of hydroxyl radicals and changes in the protective functions of cells as well as antibiofilm potential [66]. In addition, synergistic action (AgNPs + antibiotics) is related to chemical interaction, the bonding reaction between antibiotics and AgNPs, as antibiotics contain many groups like hydroxyl and amido functional groups which react with silver NPs in the chelation process [58, 69]. However, antibiotics in combination with nanoparticles can also display an antagonistic or additive effect against tested microorganisms [65, 66], as recorded in the present study. Fayaz and coauthors [69] noted a synergistic effect of antibiotics such as ampicillin, chloramphenicol, erythromycin, and kanamycin with AgNPs from *Trichoderma viride* against *E. coli*, *Salmonella Typhi*, *Micrococcus luteus* and *S. aureus*, with the greatest enhancement effect in case of the combination of ampicillin and AgNPs. Similarly, Wypij and coauthors [10] demonstrated the synergistic effect of ampicillin, kanamycin or tetracycline, and AgNPs synthesized from actinomycetes against *B. subtilis*, *E. coli*, *K. Pneumoniae*, and *P. aeruginosa*.

### Biofilm Formation Assay

**Fig. 3:**
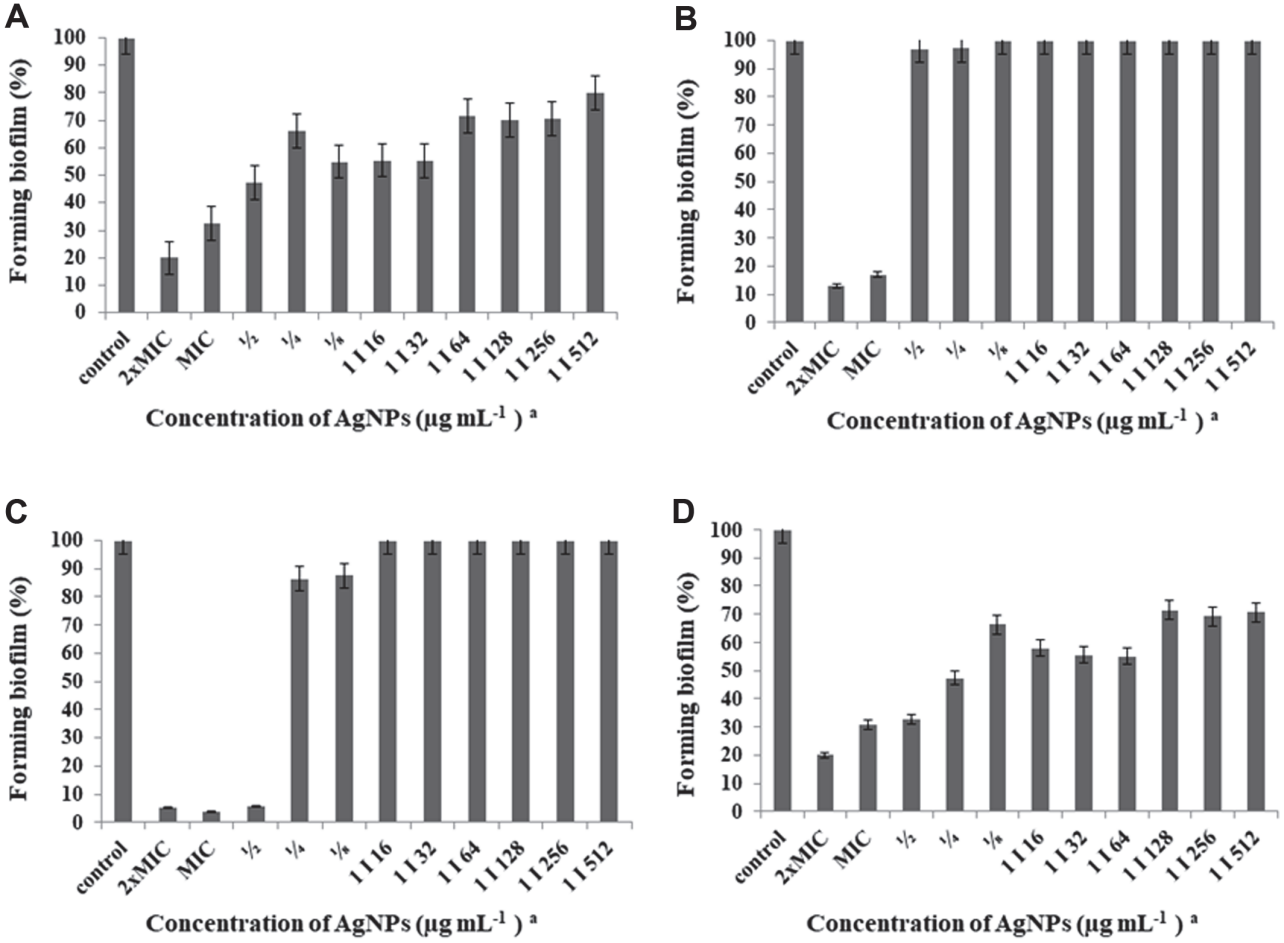
Activity of biosynthesized AgNPs against biofilm formation by (A) *S. aureus*, (B) *K. pneumoniae*, (C) *P. aeruginosa*, (D) *E. coli*.

Biofilm provides bacterial cells with effective protection against antibiotics and allows them to acquire resistance through genetic changes [3, 70, 71]. Therefore, it is important to constantly control the formation of biofilms and eliminate them in order to inhibit infection and the spread of diseases [72]. Nanotechnology offers an effective tool in a form of AgNPs against the formation of bacterial biofilms [73]. In this study, biogenic AgNPs showed a dose-dependent inhibition of the biofilm formation by both gram-positive and gram-negative bacteria and higher inhibition against the latter. Overall, biofilm formation of all bacteria was significantly inhibited at MICs and 2 × MICs (32.7-94.7% and 30.8–96.1%, respectively) with the highest inhibition in the case of *P. aeruginosa*. For *P. aeruginosa*, the formation of biofilm was also significantly inhibited at ½ MIC (16 μg/ml). Lower concentrations of Ag nanoparticles were not efficiently active against biofilm formation (Fig. 3). Recently, Singh *et al*. [11] also noticed higher inhibition of biofilm formation by gram-negative bacteria, namely *P. aeruginosa* (at MIC = 6.25 μg/ml) and *E. coli* (at MIC = 12.5 μg/ml) than gram-positive bacteria, such as *S. epidermidis* and *S. aureus* (both at MIC = 100 μg/ml). Similar to our study, they observed that biofilm production by *P. aeruginosa* and *E. coli* was significantly inhibited at sub-MIC concentrations (0.25 × MIC). It is suggested that biofilm formation is prevented by the release of Ag^+^ from the AgNPs which changes the gene expression in bacterial cells or disturbs quorum sensing in the biofilm [2, 71].

### Cytotoxicity of AgNPs

**Fig. 4:**
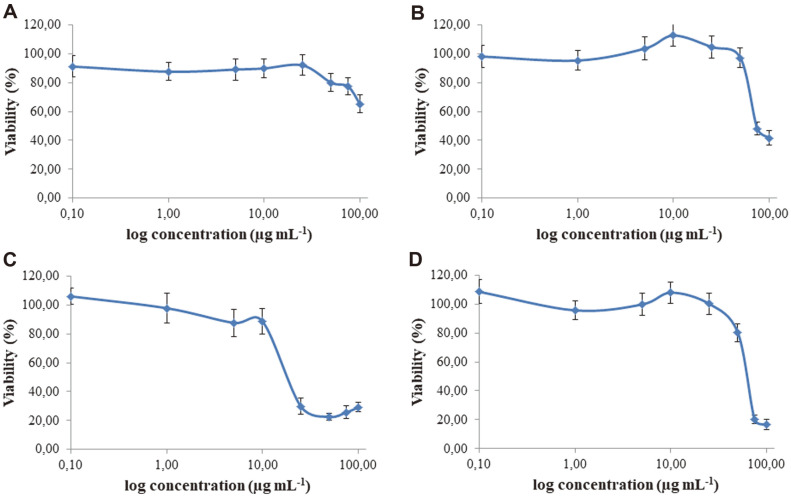
Cytotoxic activity of biosynthesized AgNPs toward A549 (A), MCF-7 (B), HepG2 (C) and A375 (D) cell lines estimated by MTT assay.

**Fig. 5:**
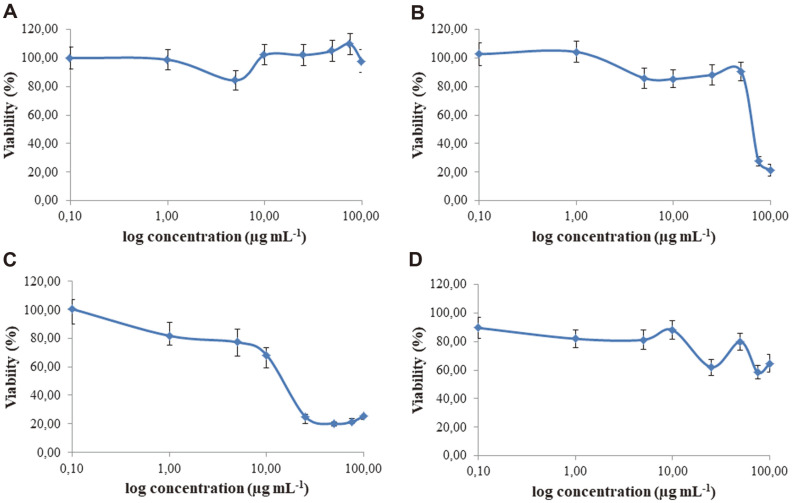
Cytotoxic activity of biosynthesized AgNPs toward A549 (A), MCF-7 (B), HepG2 (C) and A375 (D) cell lines estimated by NRU assay.

**Fig. 6:**
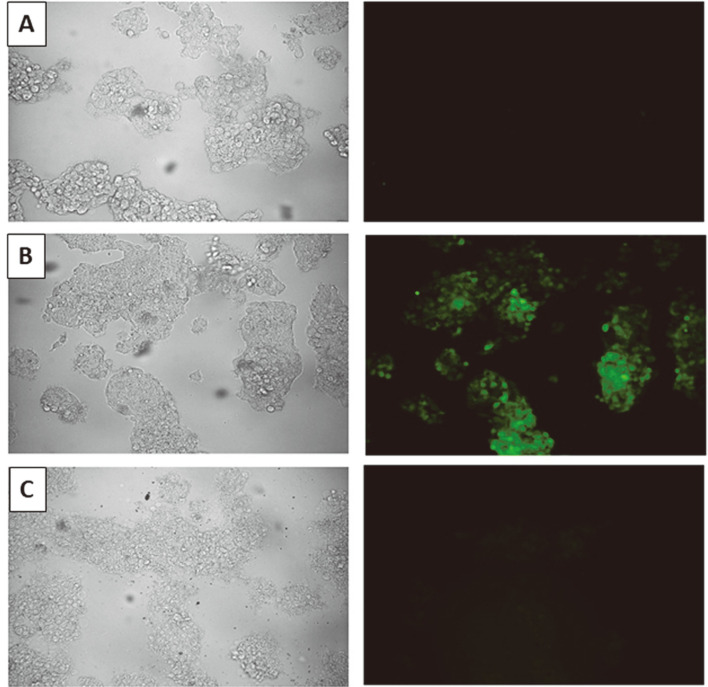
ROS generation in HepG2 cells in CM-H2DCFDA assay. Cells were incubated with vehicle control (**A**), H_2_O_2_ (**B**) or AgNPs (**C**) and observed under fluorescence microsope. Figure represents microphotographs taken after 20 min of incubation. AgNPs are represented by concentration of 100 μg/ml.

The results of in vitro study of anticancer activity of Ag nanoparticles towards MCF-7, A549, HepG2, and A375 cell lines using MTT and NR assays that measured mitochondrial activity and cell dye uptake and storage in lysosomes, respectively, are presented in Figs. 4 and 5. The HepG2 cells, among others tested, were the most sensitive to biosynthesized nanoparticles (Fig. 4). It is claimed that cytotoxicity in HepG2 cells results from damage to the mitochondrial membranes and functions that activate caspases which instigate apoptosis [74, 75]. In contrast, lung cancer cells (A549) were not sensitive to AgNPs used in the tested concentration range (Figs. 4-5, Table 3). The decrease of the viability of these cells was very low at the highest (100 μg/ml) tested dose of AgNPs by MTT assay and undetectable by NR assay. The A375 melanoma cell line was sensitive to AgNPs at concentrations above 50 μg/ml while the NR uptake ability of cells was not impaired (Figs. 4-5, Table 3). The AgNPs affected MCF-7 cells in a similar way in both assays. The IC_50_ values of AgNPs in the MTT and NR assays were 63.07 and 63.13 μg/ml, respectively (Table 3). The differences in cell response to nanosilver treatment may result from the type of cells used [76], source of bio- AgNPs, or even batch of biosynthesized AgNPs from the same source, as discussed below. The AgNPs that were previously synthesized from strain SL19 [17] showed cytotoxicity towards both cancer (HeLa) and 3T3 mouse fibroblasts. However, cancer cells were less sensitive than fibroblasts (IC_50_ of 55 and 25 μg/ml, respectively) [17]. Similar results to ours on the cytotoxicity of biogenic AgNPs to HepG2 were observed by other authors [77]. They tested AgNPs from aqueous extract of seaweed *Gracilaria corticata* and found that nanosilver at doses of 6.25 and 12.5 μg/ml effectively induced a toxic effect in HepG2 cells. Similarly, Noorbazargan *et al*. [78] presented the cytotoxicity of AgNPs biosynthesized by *Juniperus chinensis* leaf extract on MCF-7 tumor cells at a dose of 12.5 μg/ml. The most pronounced effect out of all tested cell lines was the ROS generation by biosynthesized AgNPs observed in HepG2 cells (Figs. 6 and S6-S7). No fluorescence was noted in vehicle control (negative control), while the signal was observed in cells treated with hydrogen peroxide (positive control). El-Hussein and Hamblin [79] reported generation of ROS, decrease in cell viability, and alterations in mitochondrial membrane potential of A549 cell line after treatment with AgNPs and concluded that such treatment can lead to DNA injury and cell death. Wypij *et al*. [34] assessed the capacity of different concentrations of bio-AgNPs from actinomycetes to generate intracellular ROS against MCF-7 cancer cells using DCFH-DA assay. They found that the analyzed AgNPs increased the generation of ROS compared to untreated control cells.

### Genotoxicity of AgNPs

AgNPs are probably responsible for biochemical and molecular changes related to genotoxicity in cells [80]. Donaldson *et al*. [81], suggested that genotoxicity of AgNPs can be the direct result of NP interaction with DNA or an indirect result due to the generation of ROS or ion release leading to DNA damage. One of the mechanisms of genotoxicity is mutagenicity which can be assessed by bacterial reverse mutation test [80]. In the present study, AgNPs at a dose of 3 μg/ml or higher denotes their low toxicity level and test doses did not affect reverse mutation in cells of *Salmonella Typhimurium* strain TA98. Thus, the genotoxicity of AgNPs in the test concentration range was not recorded. The reduction of the revertant frequency in *Salmonella Typhimurium* strain was found at ≥ 3 μg of AgNPs per plate (Table 4).

### Catalytic Degradation of Organic Dye

The catalytic degradation of methyl orange with NaBH_4_ and AgNPs is shown in Fig. S8. The reduction of MO with NaBH_4_, but without AgNPs as a catalyst, occurred at a very slow rate. AgNPs possess high catalytic properties compared to other metallic nanoparticles [82]. They can be utilized for the degradation of organic dyes, such as azo dyes, that are widely used in textiles, plastics, and medicine, and are one of the major pollutants in the environment [83]. In our work, there was no shift in absorbance for MO solution which was not exposed to AgNPs, as a nanocatalyst. Similarly, Cyril *et al*. [82], reported that 30 μl of nanocatalyst containing 4.9 μg of AgNPs showed a reduction of the peak intensity at the wavelength of 464 nm. This is a consequence of the reduction of the azo (–N=N–) bond in methyl orange dye to corresponding colorless amine (–NH–NH–) with the addition of AgNPs. Overall, nanosilver is believed to be a stable photocatalyst for the efficient degradation of organic compounds, including dyes, especially at ambient temperature and with the use of visible light [84].

### Catalytic Activity of AgNPs

The photocatalytic degradation of MO was evaluated by using different concentrations of AgNPs at specific time intervals, as shown in Fig. S9. A gradual decrease in UV-Vis absorbance over time was observed, confirming the degradation of the dye in the presence of nanoparticles. Moreover, we observed that the rate of MO reduction increased with increasing concentration of the biogenic silver nanoparticles (Fig. S9). Similar observations were reported by other authors [85, 86]. The absorbance peak of MO dye was slowly decreased with time up to 4.5 h of incubation with AgNPs from leaf extract of *Justicia adhatoda* [86]. Similar to our study, Bhakya *et al*. [85] reported photocatalytic activities of biogenic AgNPs from Helicteres isora that were tested in the concentration range of 20–100 μg/ml in time. The results showed that the reduction of MO increased with the increasing AgNP concentration. Photocatalytic activity of silver nanoparticles in sunlight was explained as the reduction of MO induced by photons of sunlight striking the surface of silver nanoparticles [86]. Nanoparticles support the electron relay donor-acceptor and behave as a substrate for the electron transfer process. Therefore, AgNPs are efficient catalysts by transfer of electron reaction [85, 87].

To sum up, an acidophilic actinomycete of *Pilimelia* sp. strain SL19 was found to be an efficient producer of spherical, small, stable, and protein-capped silver nanoparticles. Such rare genera of actinomycetes isolated from harsh environments are particularly considered as a potential source of unique bioactive compounds or enzymes that can be involved in the biosynthesis process of AgNPs. The natural origin proteins may be important agents responsible for the reduction of Ag^+^ to AgNPs, the stabilization of nanoparticles, and their bioactivity. Nanosilver fabricated using actinobacterial SL19 strain seems to be a promising antibacterial agent, mainly towards gram-negative bacteria, as they significantly inhibited bacterial growth and showed biocidal and antibiofilm formation properties as well as enhanced antibiotic activities, especially in the case of tetracycline. These bio-AgNPs presented significant cytotoxicity, especially against the HepG2 cell line. Therefore, AgNPs from the actinobacterial SL19 strain may also be promising anticancer agents that could be considered in medicine in the future. Biosynthesized AgNPs can also be seen in the industry as a reduction agent for the elimination of toxic dyes. However, it should be highlighted that each batch of biogenic nanoparticles may display different properties, as proved in the studies on AgNPs from actinomycete SL19 strain. Finally, further studies are needed to figure out the mechanism of action of biogenic nanosilver, especially in the context of the presence of capping proteins, against both bacteria and cancer cell lines.

## Supplemental Materials

Supplementary data for this paper are available on-line only at http://jmb.or.kr.

## Figures and Tables

**Table 1:** Minimum inhibitory concentrations (MICs) and minimum biocidal concentrations (MBCs) of AgNPs and antibiotics (μg/ml).

Tested microorganism	MIC of AgNPs	MBC of AgNPs	MIC of antibiotics				MBC of antibiotics			

			AMP	S	TE	K	AMP	S	TE	K
*E. coli* ATCC 8739	64	64	4	16	0.5	16	4	64	>2048	16
*K. pneumoniae* ATCC 700603	256	256	2048	4	16	128	>2048	4	512	>2048
*P. aeruginosa* ATCC 1014	32	32	512	64	8	128	512	64	32	128
*S. aureus* ATCC 6538	256	512	0.064	4	0.016	4	0.064	4	>2048	4

MIC minimum inhibitory concentration, MBC minimum biocidal concentration, AMP ampicillin, K kanamycin, TE tetracycline, S streptomycin

**Table 2:** Fractional inhibitory concentration index (FICI) determined for AgNPs from SL19 strain and antibiotics against bacteria.

Tested microorganism	FICI index			

	Streptomycin + AgNPs	Kanamycin+ AgNPs	Ampicillin+ AgNPs	Tetracycline+ AgNPs
*Staphylococcus aureus* ATCC 6538	1.0	1.0	1.0	1.0
*Escherichia coli* ATCC 8739	0.5	2.0	1.0	0.25
*Pseudomonas aeruginosa* ATCCC 10145	0.25	1.0	1.0	0.25
*Klebsiella pneumoniae* ATCC 700603	0.06	2.0	2.0	0.123

≤ 0.5 synergism (at least 4 times reduction of dose); > 0.5- 1.0 non-synergistic or additive effect; ≥ 1.0 – 2.0 indifferent effect [32]

**Table 3:** IC50 values (μg/ml) for AgNPs obtained by MTT and NRU assay.

Cell line	MTT	NR
A549	>100	>100
MCF-7	63.07	63.13
HepG2	14.58	12.57
A375	55.41	>100

**Table 4:** Mutagenicity of AgNPs in *Salmonella Typhimurium* TA98 test strain.

Dose (μg/plate)	Number of colonies/plate (mean ± SD)
Negative control	29 ± 4
0.15	27 ± 2
0.25	28 ± 4
0.5	24 ± 3
1.0	21 ± 3
1.5	14 ± 2
3.0	T
6.0	
12.5	
25.0	
37.5	
50.0	
100.0	
Positive control	962 ± 17^a^

Notes: SD represents standard deviation; T denotes toxicity detected at this and higher doses either as a reduction in the spontaneous frequency or a thinning of the background lawn.

^a^*p* < 0.05 vs. control.
