# Supplementary material for: Novel Antibacterial, Cytotoxic and Catalytic Activities of Silver Nanoparticles Synthesized from Acidophilic Actinobacterial SL19 with Evidence for Protein as Coating Biomolecule
Source: J Microbiol Biotechnol. 2022 Aug 22;32(9):1195–208. doi: 10.4014/jmb.2205.05006 (PMC9628977; doi:10.4014/jmb.2205.05006)
Supplement: Supplementary file 1 [file jmb-32-9-1195-supple.pdf]

Supporting Information

Novel antibacterial, cytotoxic and catalytic activities of silver nanoparticles synthesized from acidophilic actinobacterial SL19 with evidences for protein as coating biomolecule

Magdalena Wypij[1*], Maciej Ostrowski[2], Kamil Piska[3], Katarzyna Wójcik-Pszczoła[3], Elżbieta Pękala[3], Mahendra Rai[1,4], Patrycja Golińska[1]

[1]Department of Microbiology, Nicolaus Copernicus University, Torun, Poland; [2]Department of Biochemistry, Nicolaus Copernicus University, Torun, Poland; [3]Department of Pharmaceutical Biochemistry, Faculty of Pharmacy, Jagiellonian University Collegium Medicum, Krakow, Poland; [4]Nanobiotechnology Laboratory, Department of Biotechnology, SGB Amravati University, Amravati, India

*Corresponding author: mwypij@umk.pl

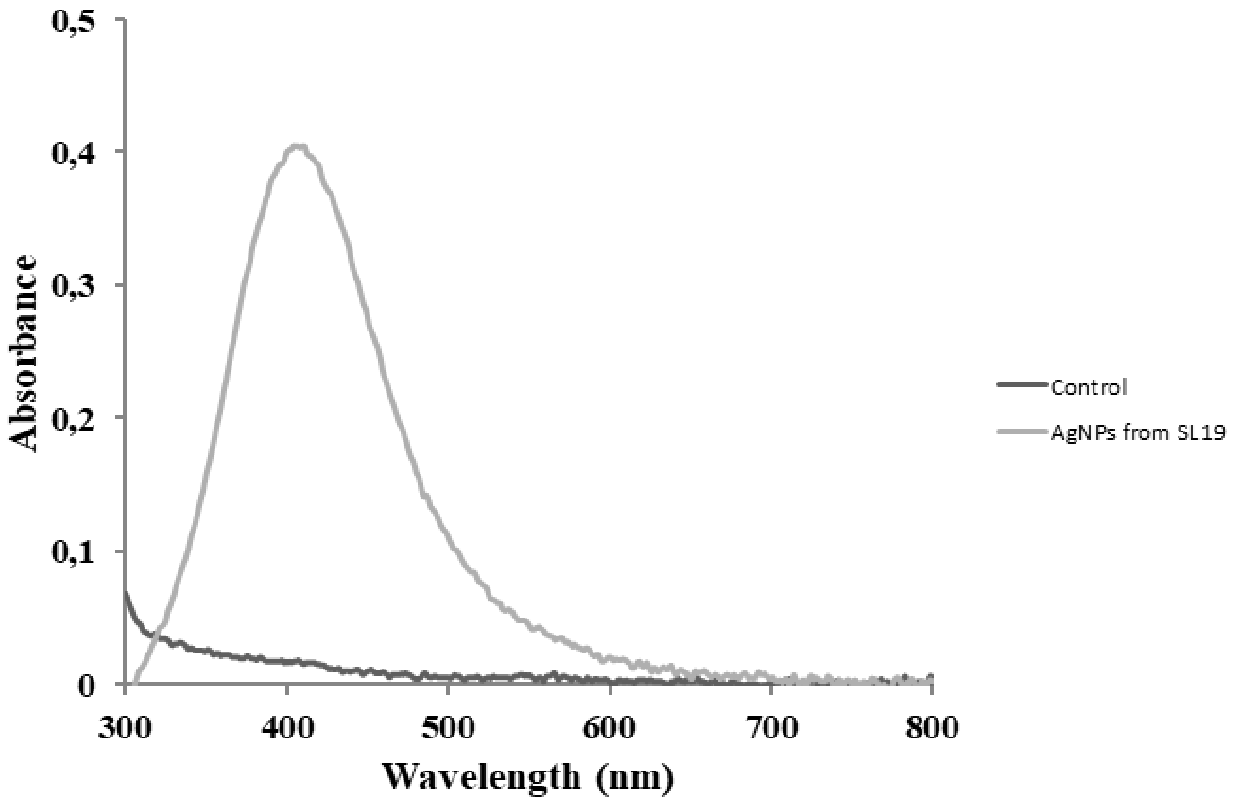

Figure S1: UV-Vis spectrum of biosynthesized AgNPs

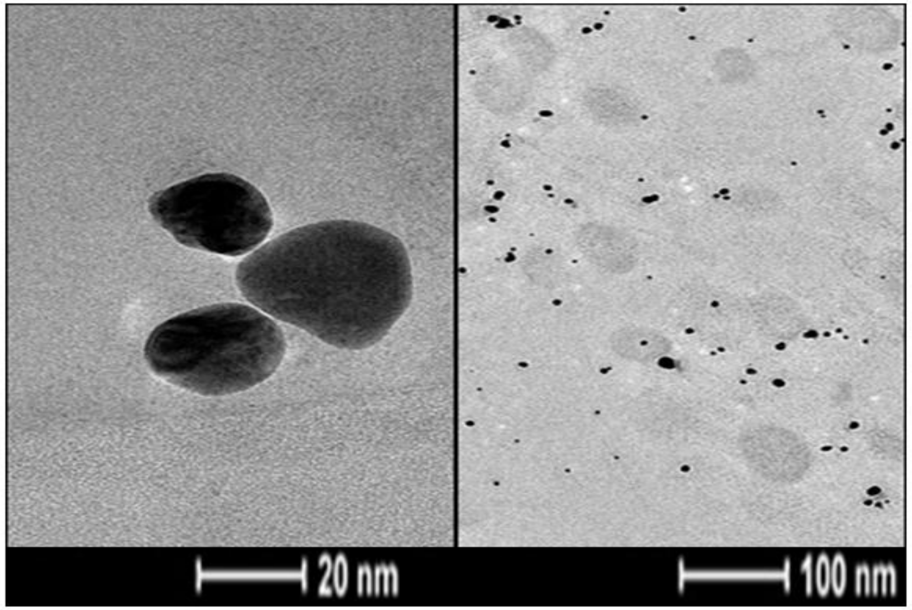

Figure S2: TEM of biosynthesized AgNPs

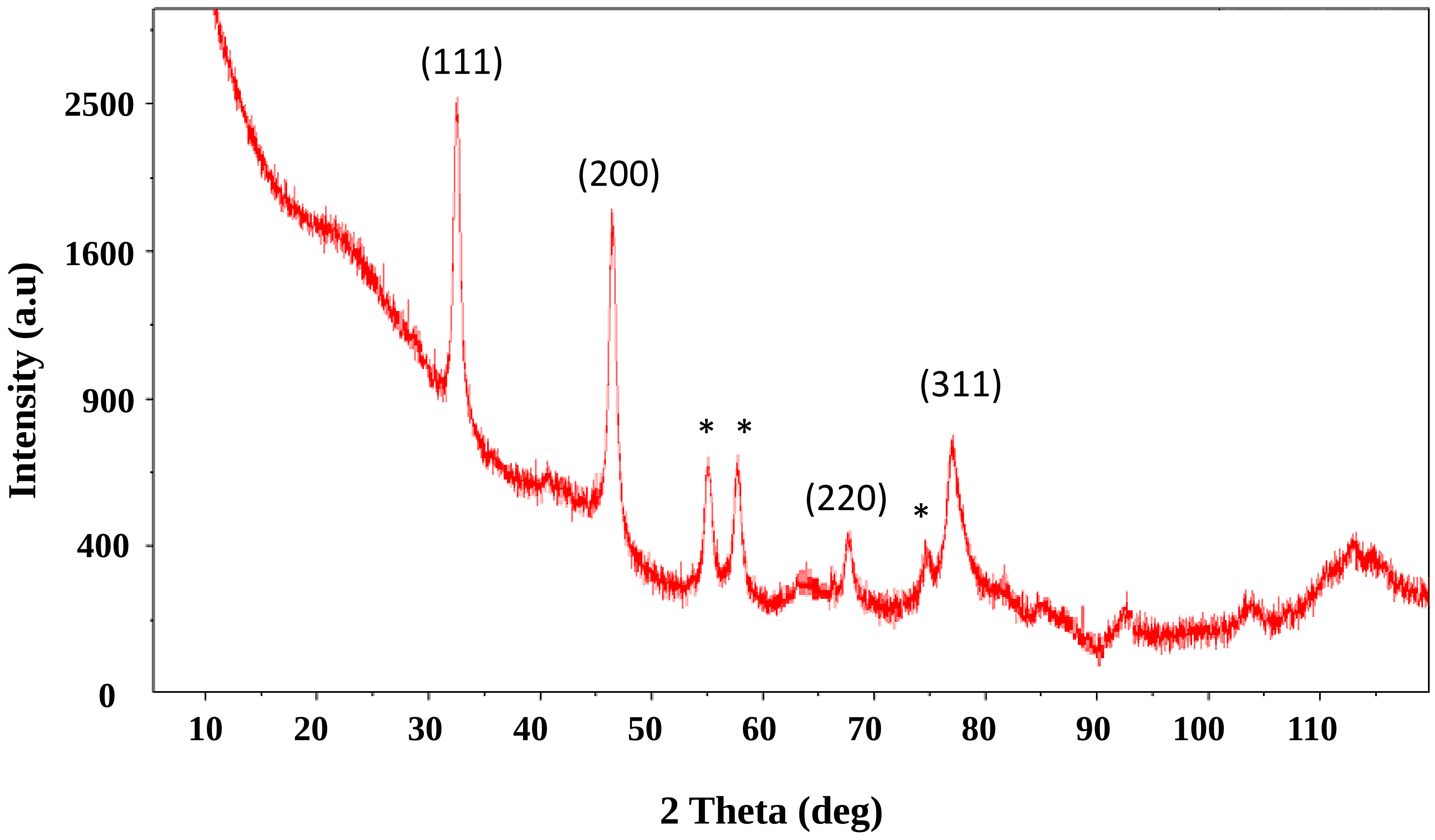

Figure S3: X-Ray Diffraction spectroscopy analysis

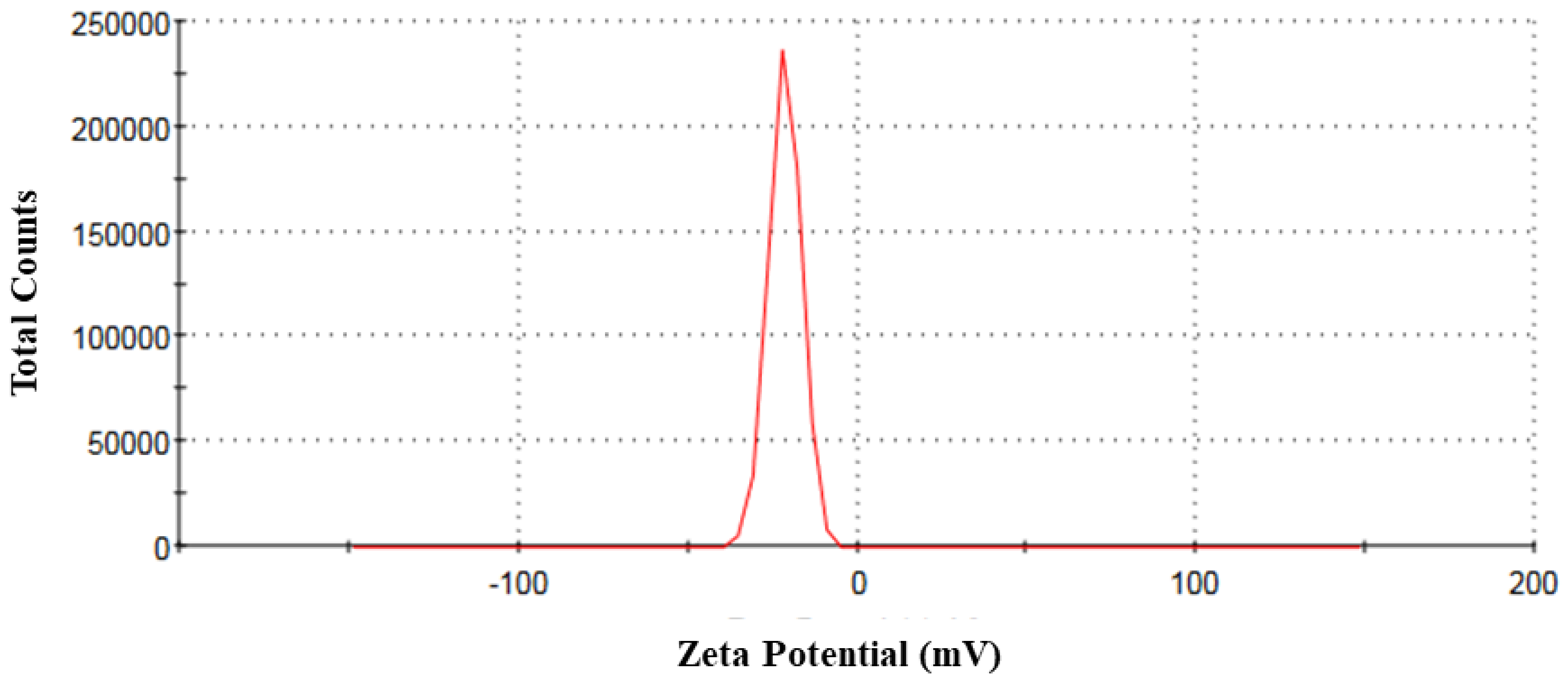

Figure S4: Zeta potential graph of biosynthesized AgNPs

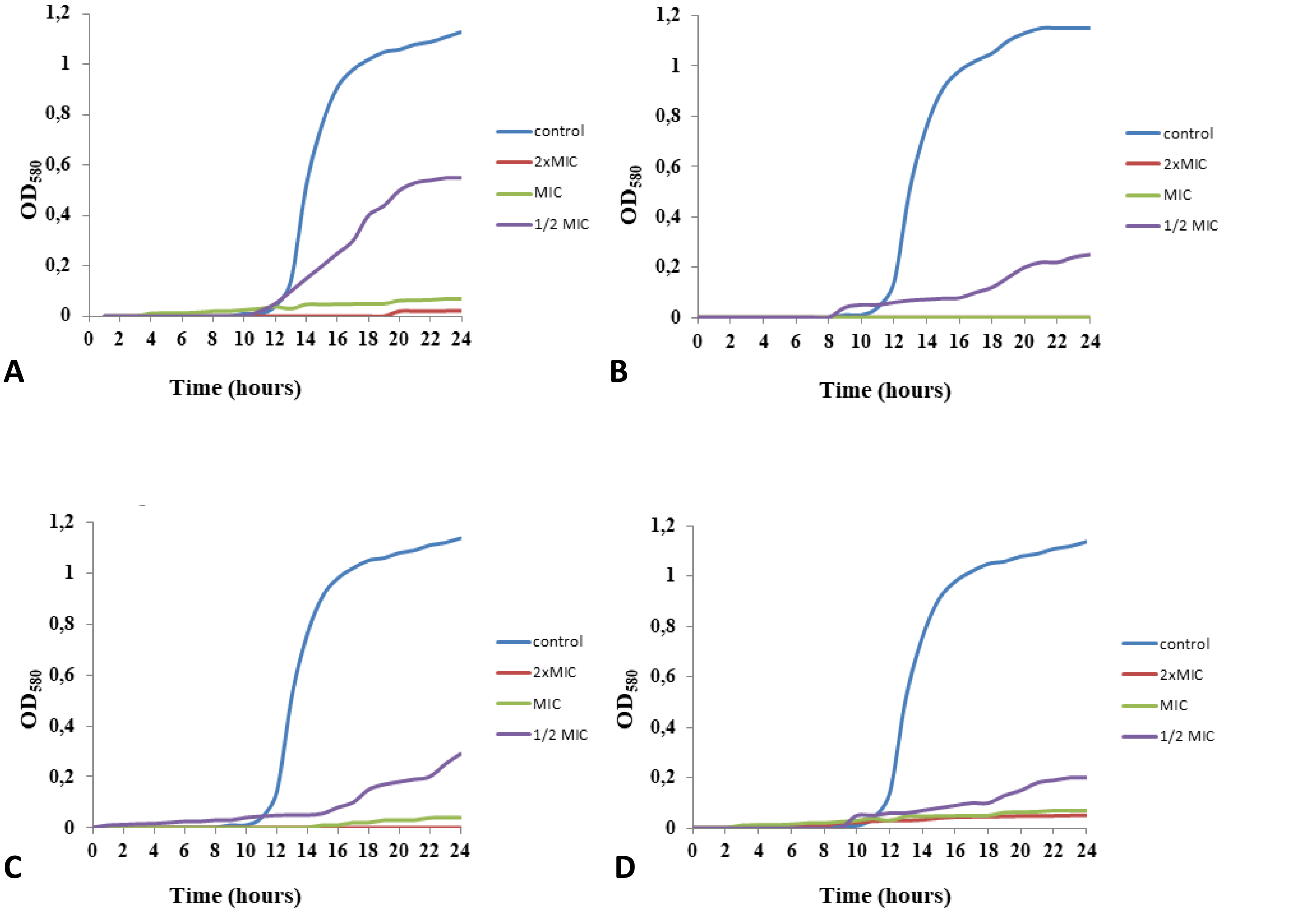

Figure S5: Effect of AgNPs on bacterial growth. *S. aureus* (**A**), *K. pneumoniae* (**B)**, *P. aeruginosa* (**C**), *E. coli* (**D**)

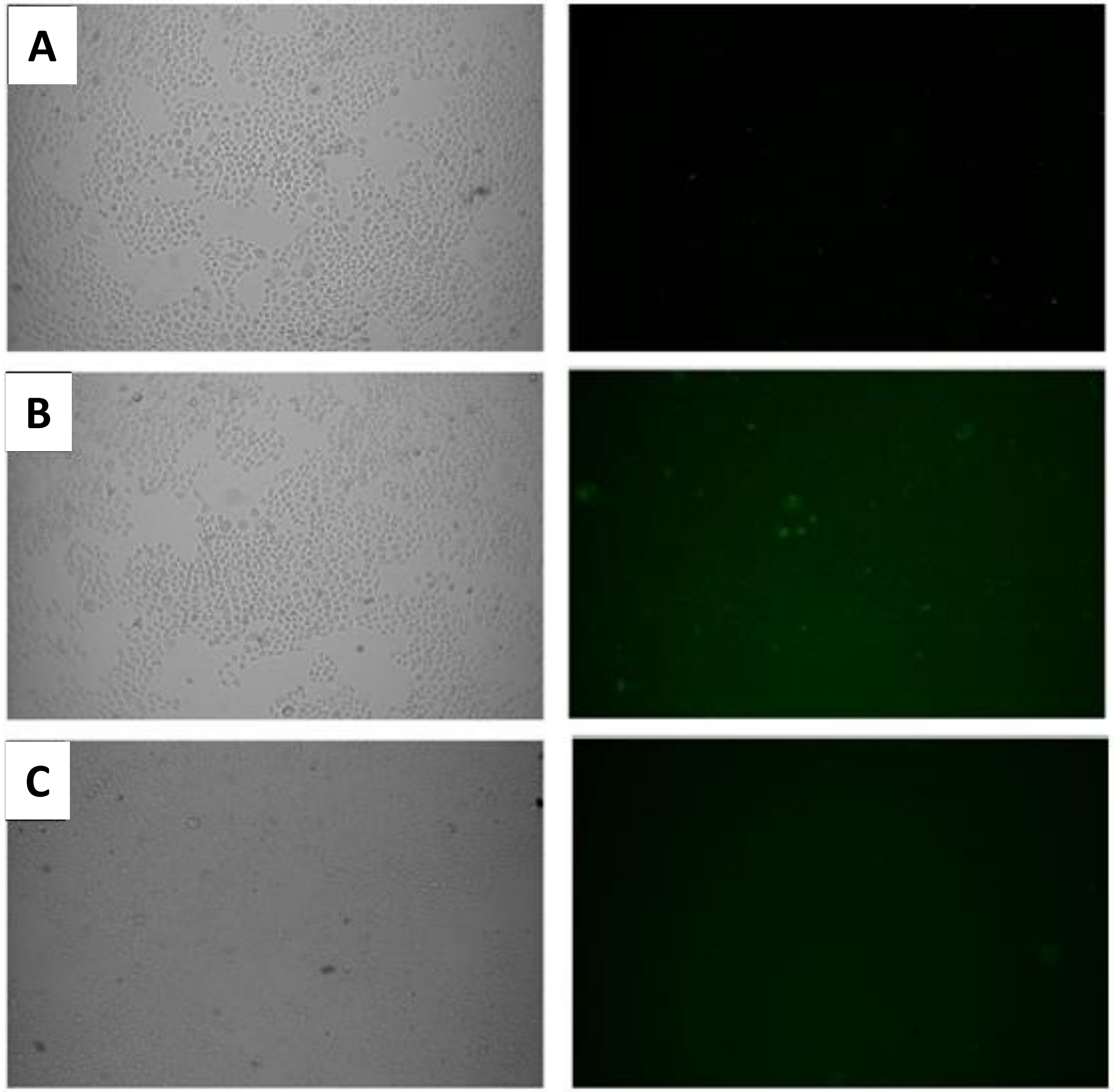

Figure S6: ROS generation in A549 cells in CM-H2DCFDA assay. Cells were incubated with vehicle control (**A**), H_2O_2 (**B**) or AgNPs (**C**) and observed under fluorescence microsope Leica DMiL LED Fluo. Figure represents microphotographs taken after 20 minutes incubation. AgNPs are represented by concentration of 100 μg mL^{-1}

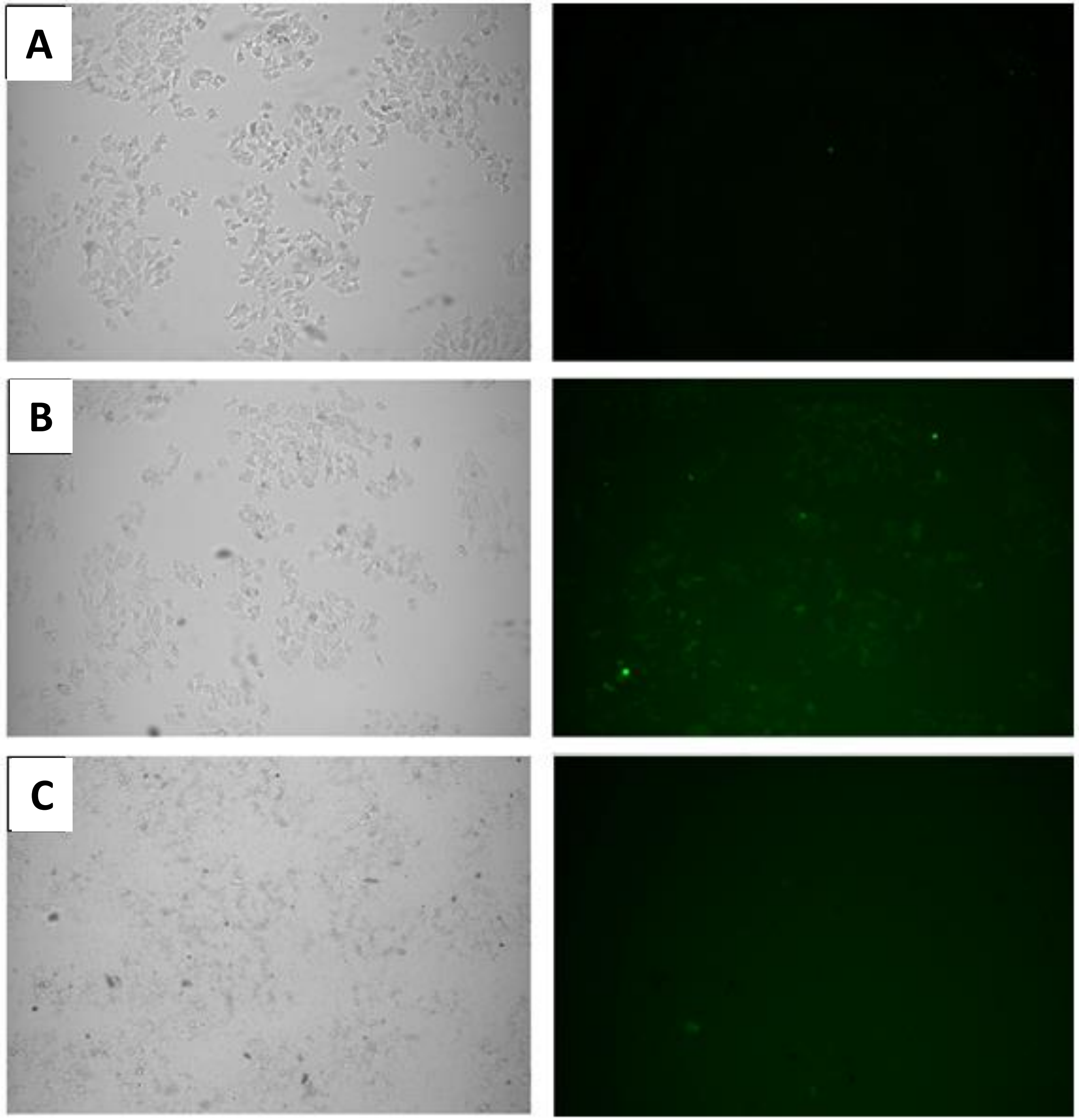

Figure S7: ROS generation in MCF-7 cells in CM-H2DCFDA assay. Cells were incubated with vehicle control (**A**), H_2O_2 (**B**) or AgNPs (**C**) and observed under fluorescence microsope Leica DMiL LED Fluo. Figure represents microphotographs taken after 20 minutes incubation. AgNPs are represented by concentration of 100 μg mL^{-1}.

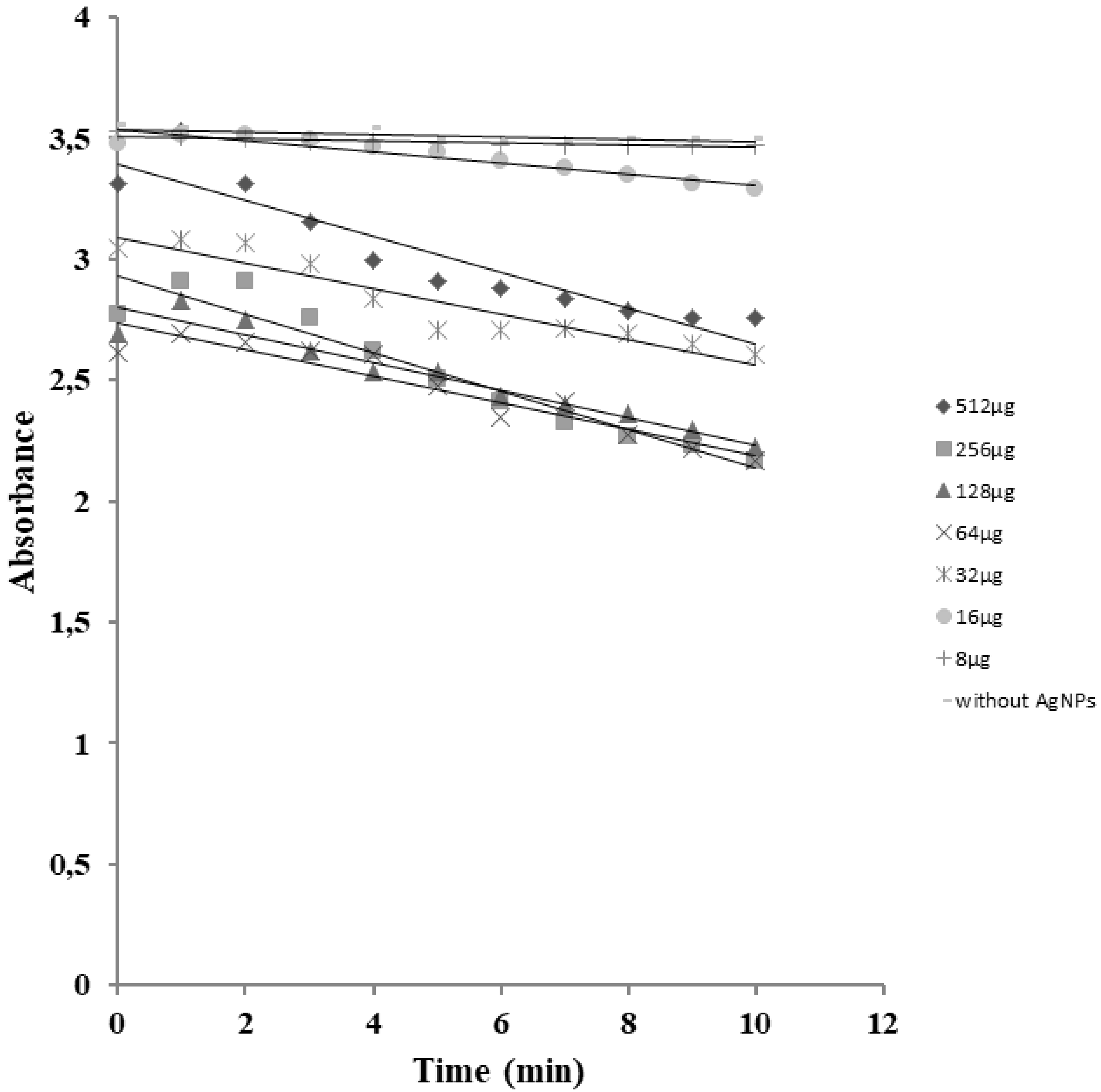

Figure S8: The catalytic reduction of methyl orange (MO) by using AgNPs

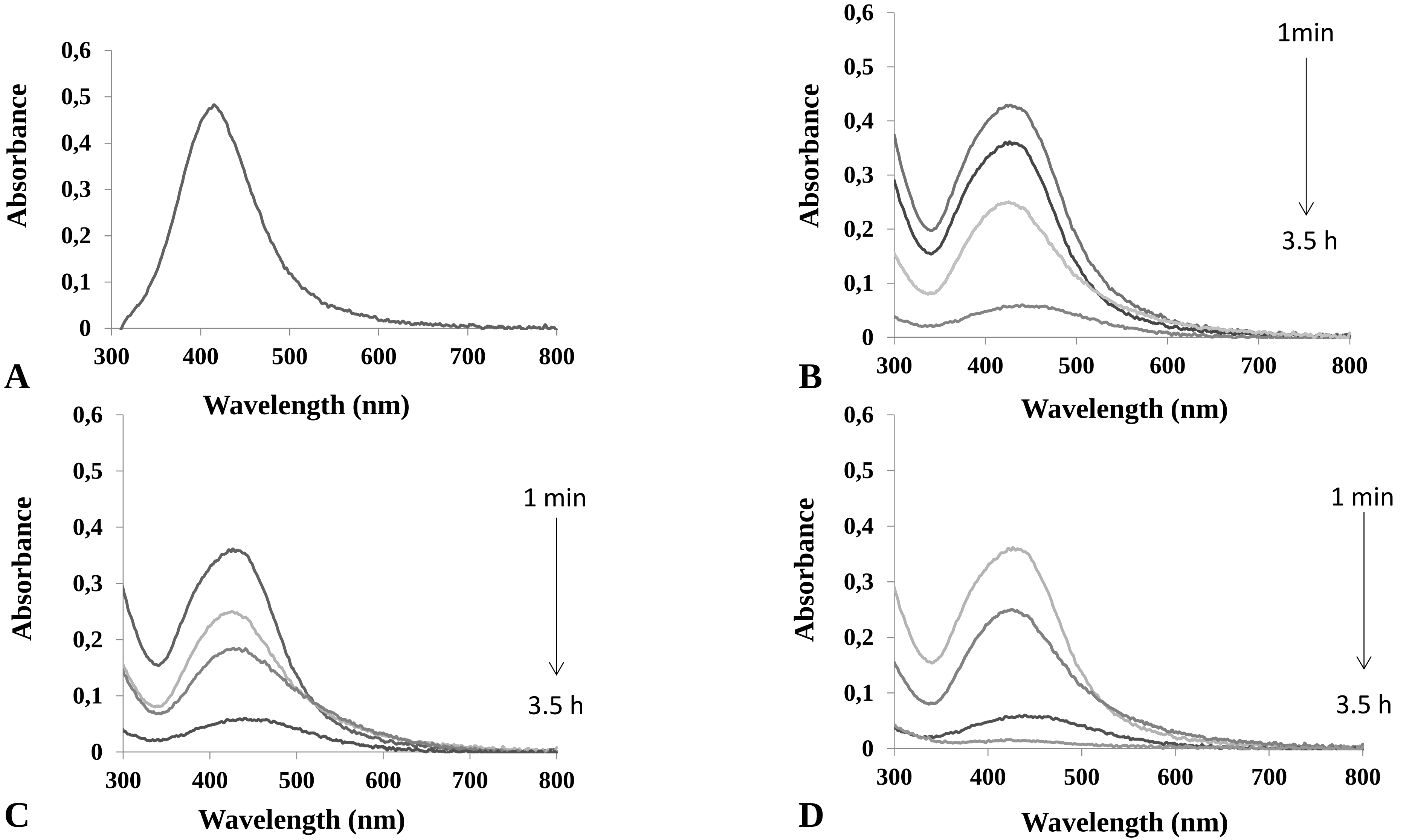

Figure S9: UV-visible absorption spectra of degradation of MO in the presence of AgNPs
(A) MO in absence of AgNPs; (**B)** 32 ug mL^{-1} **(C)** 64 ug mL^{-1} **(D)** 256 ug mL^{-1}

Table S1: Identification of proteins associated with silver nanoparticles synthesized from actinobacterial strain SL19.

**Band number**	**Accession**	**Description**	**Taxonomy**	**Coverage (%)**	**Molecular weight (kDa)**	**Calculated pI**	**Score**
**01**	WP_108997678.1	Hypothetical protein, partial	Escherichia coli	43.0	41.0	8.47	3160
	WP_031376623.1	porin OmpF	Pantoea sp. 3.5.1	52.0	Unknown	4.59	2170
**02**	WP_008643071.1	MULTISPECIES: porin	Cupriavidus	59.0	38.0	8.81	3501
	WP_008645123.1	Porin	Cupriavidus sp. HMR-1	48.0	Unknown	9.08	2315
	WP_009241956.1	MULTISPECIES: porin	Ralstonia	42.0	Unknown	9.42	1116

Table S2: MBC/MIC ratio of AgNPs and antibiotics.

	Ratio MBC/MIC				
	**AgNPs**	**Streptomycin**	**Kanamycin**	**Ampicillin**	**Tetracycline**
***Escherichia coli* ATCC 8739**	1	4	1	1	>4096[a]
***Klebsiella pneumoniae* ATCC 700603**	1	1	>16[a]	>1[a]	32
***Pseudomonas aeruginosa* ATCC 1014**	1	1	1	1	4
***Staphylococcus aureus* ATCC 6538**	2	1	1	1	>128000[a]

[a]; MBC of antibiotic >2048 µg mL^{-1}
